# 

**Published:** 1843-03-04

**Authors:** 


// THE MEDICAL EXAMINER,

**And Retrospect of the Medical Sciences.**

EDITED BY

MEREDITH CLYMER, M.D.

Lecturer on the Institutes of Medicine, Physician to the Pennsylvania Institution for the Blind, Fellow of the College of Physicians, &c. &c.

VOL. VI.] PHILADELPHIA, MARCH 4, 1843. [No. 4.

